# Natural Theology

**Published:** 1853-05

**Authors:** 


Art. XVI.—*Natural Theology.* A Valedictory Lecture, delivered to the class of students in the Medical College of Ohio, in the Department of Chemistry, February 24th, 1853, by John Locke, M. D., Professor of Chemistry and Pharmacy.

Professor Locke has distinguished himself in several walks of Natural Science, and ranks among the profound and original thinkers of his country. He has impressed himself upon the science of the age, and will be mentioned respectfully in coming time as one who labored successfully for its advancement. Whether it will prove that he has made any substantial additions to the science of Natural Theology by the discourse before us, we shall not undertake to say. It is not often that men excel in departments of human knowledge so wide apart as physics and theology. The great mind of Newton failed to master both domains, and by the weakness displayed in his theological speculations, it has been said he afforded to other men some sort of compensation for his incomparable superiority to them in astronomy. Dr. Locke may not have materially strengthened the argument of Paley and Brougham, but he has brought forward new facts and illustrations, and the presentation of the subject to his pupils in the light in which he has studied it, can hardly fail to impress their minds with its elevation and importance.—Medicine, as he justly observes, "is a study of the laws of eur Creator," and it should be the aim of every teacher to exhibit our profession in these its highest and most sacred relations.

"In a certain sense," he remarks, "these halls are consecrated, not only to science, but to the purposes of religion and divinity. God is truth, and all truth, especially physical truth, is a part of himself, and a part of his revelation to man. In this sense, the demonstration table becomes a sacred altar, and this hall of science, a temple dedicated to his service. Experiments are the visible evidence of his presence, of his wisdom, and of his power. It is true, the circumstances do not permit us, currently, to apply all of these things to the purposes of ethical divinity, nor would this course be well timed, towards the audience, at each lecture. But at the close of a great work of instruction, we may be permitted to say, it is God's work, and not ours. We may be permitted to thank him for the manifestations of himself, which he has made through these works; for the capacity which he has given us, for comprehending his glorious and wise laws, and for the power which he has given, of applying the knowledge derived from him, to the benevolent purpose of relieving the sufferings of our fellow beings."

Dr. Locke has been long a teacher of youth, and such words come with a peculiar grace from one who has had so much experience in the realities and the vanities of life.

